# Genome-wide profiling of humoral immunity and pathogen genes under selection identifies immune evasion tactics of *Chlamydia trachomatis* during ocular infection

**DOI:** 10.1038/s41598-017-09193-2

**Published:** 2017-08-29

**Authors:** Harry Pickering, Andy Teng, Nkoyo Faal, Hassan Joof, Pateh Makalo, Eunice Cassama, Meno Nabicassa, Anna R. Last, Sarah E. Burr, Sarah L. Rowland-Jones, Nicholas R. Thomson, Chrissy h. Roberts, David C. W. Mabey, Robin L. Bailey, Richard D. Hayward, Luis M. de la Maza, Martin J. Holland

**Affiliations:** 1 Clinical Research Department, Faculty of Infectious and Tropical Diseases, London School of Hygiene and Tropical Medicine, London, WC1E 7HT United Kingdom; 2 ImmPORT Therapeutics, Inc./Antigen Discovery Inc., 1 Technology Dr., Suite E309, Irvine, CA 92618 United States; 3 Disease Control and Elimination Theme, Medical Research Council The Gambia Unit, Fajara Banjul, The Gambia; 4 Programa Nacional de Saúde de Visão, Ministério de Saúde Publica, Bissau, Guinea-Bissau; 5 Department of Pathogen Molecular Biology, London School of Hygiene and Tropical Medicine, London, WC1E 7HT United Kingdom; 6 Pathogen Genomics, Wellcome Trust Sanger Institute, Wellcome Trust Genome Campus, Hinxton, United Kingdom; 7 Institute of Structural and Molecular Biology, Birkbeck & University College London, Malet Street, London, WC1E 7HX United Kingdom; 8 Department of Pathology and Laboratory Medicine, Medical Sciences I, Room D440, University of California, Irvine, CA 92697-4800 United States

## Abstract

The frequency and duration of *Chlamydia trachomatis* (Ct) ocular infections decrease with age, suggesting development of partial immunity. However, there is a lack of clear correlates of immunity to Ct infection in humans. We screened sera from a cohort of Gambian children followed for six-months against a Ct-proteome microarray. At genome sequence level, we detected signatures of selection from a population of ocular Ct isolates from Guinea-Bissau. Together these approaches allowed us to highlight the focus of humoral responses and hypothesise new modes of pathogen immune evasion. Children who were susceptible to frequent and/or prolonged Ct infection had a less focussed antibody response, including preferential recognition of forty-two antigens. There was evidence of positive and purifying selection across the genome, but little balancing selection. In contrast, most antigens that were associated with susceptibility were under neutral selection. These data suggest an evasion strategy in which Ct presents a large panel of irrelevant antigens to the immune system to block or misdirect protective responses. Development of a focused immune response, possibly induced through vaccination, may be an effective strategy to promote protection to Ct infection.

## Introduction

Ocular infection with *Chlamydia trachomatis* (Ct) causes trachoma, the leading infectious cause of blindness^1^. Both ocular Ct infection and active disease prevalence decline from their peaks in pre-school children (one to four years old) to older children (five to fourteen years old) and from this group to adults (fifteen years or older)^2, 3^. This suggests that partial Ct immunity develops with increasing age in endemic communities, notwithstanding reduced exposure to Ct with increasing age^4^.

Conjunctival Ct infection induces a strong pro-inflammatory response marked by production of cytokines^5^, recruitment of neutrophils, macrophages and NK-cells^6, 7^. Induction and proliferation of CD4^+^ T-cells and production of interferon-gamma (IFNγ) have been implicated in successful resolution of infection in animal models and human infections^8–11^. Ocular Ct infection additionally induces local and systemic antibodies^12^. Neutralising antibodies against Ct have been demonstrated in animal models^13^ and *in vitro* ^14^. Paradoxically in a longitudinal study from The Gambia, higher IgG responses against the immunodominant major outer membrane protein (MOMP) were associated with higher rates of infection and higher titres increased the associated risk^15^. Ocular Ct infection clearly induces a strong humoral immune response, but its role in protection or pathology in humans *in vivo* remains unclear.

Screening whole-proteome arrays has become an effective method to describe the complete profile of antibody responses in infection and disease^16^. Studies utilising these proteome arrays have highlighted some common themes in humoral immune targets of human pathogens including functions in protein binding and catalytic activities, early or late expression in the developmental cycle and membrane localisation^17^.

Genome-wide analyses of the type or mechanisms of selection have been conducted on a number of human pathogens and identified known and novel targets that were under immune selective pressures. Studies in many human pathogens, including *P. falciparum* ^18, 19^, *Helicobacter pylori* ^20^, *Staphylococcus aureus* ^21^ and *Streptococcus pneumonia* ^22^, have highlighted cellular and humoral immune targets, cell surface proteins and known host-interactors as overrepresented in genes under both balancing (selection and maintenance of multiple alleles) and positive selection (selection of advantageous alleles).

Until recently, the number of sequenced Ct genomes has been low and they have been derived from specimens that were collected from ecologically disparate sites over a timespan >50 years (Harris *et al*.^23^). A study by Thomson *et al*.^24^ utilizing 3 genomes, more recent studies by Joseph *et al*.^25, 26^ utilising 12 and 32 genomes respectively followed by Borges *et al*.^27^ (n = 59 genomes), identified positive selection in known host-interactors that were either surface-exposed or secreted into the host cytosol. Most recently, Hadfield *et al*. analysed 563 Ct genomes^28^. Similar to the prior studies, they focussed on global diversity of the species and its evolutionary history, rather than within-population dynamics. In trachoma, previous studies have focused on the population genetics of *ompA*, which encodes the immunodominant MOMP. Two populations of Ct sequences from trachoma-endemic communities in The Gambia^29^ and Tanzania^30^ suggested *ompA* was under purifying (selection against deleterious alleles) and positive selection; similar variation in selection pressure has been found in urogenital Ct sequences^31^. A lack of balancing selection in this immunodominant antigen, which is a target of neutralising antibodies, is in contrast to other pathogens and highlights the need for further population-based studies of Ct-genes under selection to better understand the interactions between Ct and the host immune system.

We used sera, collected from Gambian children at the baseline point of a six-month longitudinal cohort, to screen a protein microarray of 894 genomic ORFs from serovar D Ct^32^. The complete profile of responses for each sample was determined and used to investigate the differential recognition of individual antigens and estimate the diversity and evenness of the antibody response associated with the frequency and duration of Ct infection. A population of 126 complete genome sequences of ocular Ct samples obtained from discrete communities of four Bijagos Islands (Guinea-Bissau) collected in a single survey^33, 34^ was used in tests of population genetic selection. Genome-wide evidence of selection and genome-wide screening of the antibody response in the context of susceptibility to ocular infection observed over a 6-month period were overlaid. This enabled us to identify new targets of humoral immunity and we uncovered two complementary immune evasion tactics that may support Ct survival and promote recurrent infection.

## Results

### Immunity defined by susceptibility to infection

After normalisation and filtering to remove infrequently recognised antigens, responses of 90 individuals covering 441 antigens were included in the analysis. Individuals were divided into those resistant or susceptible based on observed median duration of infections over the six-month study period (Supplementary Figure 1). The study-wide median duration of infection was 2 weeks (or one visit). Those with no infections or short duration infections were combined (≤2 weeks; resistant) and compared to those with long duration infections (>2 weeks; susceptible). The demographic similarity of resistant and susceptible individuals indicated that history of ocular Ct exposure was similar (Table 1).

**Table 1:** Age, gender and village membership in resistant and susceptible groups screened on the micro-array.

	Resistant	Susceptible	p-value
**Number**	60	30	NA
**Age in years (95% CI)**	9.00 (2.00–11.53)	8.00 (1.73–12.00)	0.413
**Female (n [%])**	24 (40.00)	13 (43.33)	0.762
**Village (n [%])**			<0.001
**−1**	7 (11.67)	19 (63.33)	
**−2**	2 (3.33)	0 (0.00)	
**−4**	5 (8.33)	1 (3.33)	
**−6**	11 (18.33)	3 (10.00)	
**−7**	27 (45.00)	7 (23.33)	
**−8**	8 (13.33)	0 (0.00)	

Association with resistance or susceptibility to infection was determined using a generalised linear model. For age, numbers in parentheses are the 95% confidence intervals around the median. For gender and village, numbers in parentheses are the percentage. No individuals from villages 3 and 9 were screened on the micro-array.

### Diversity of the antibody response and infection susceptibility

**Figure 1:**
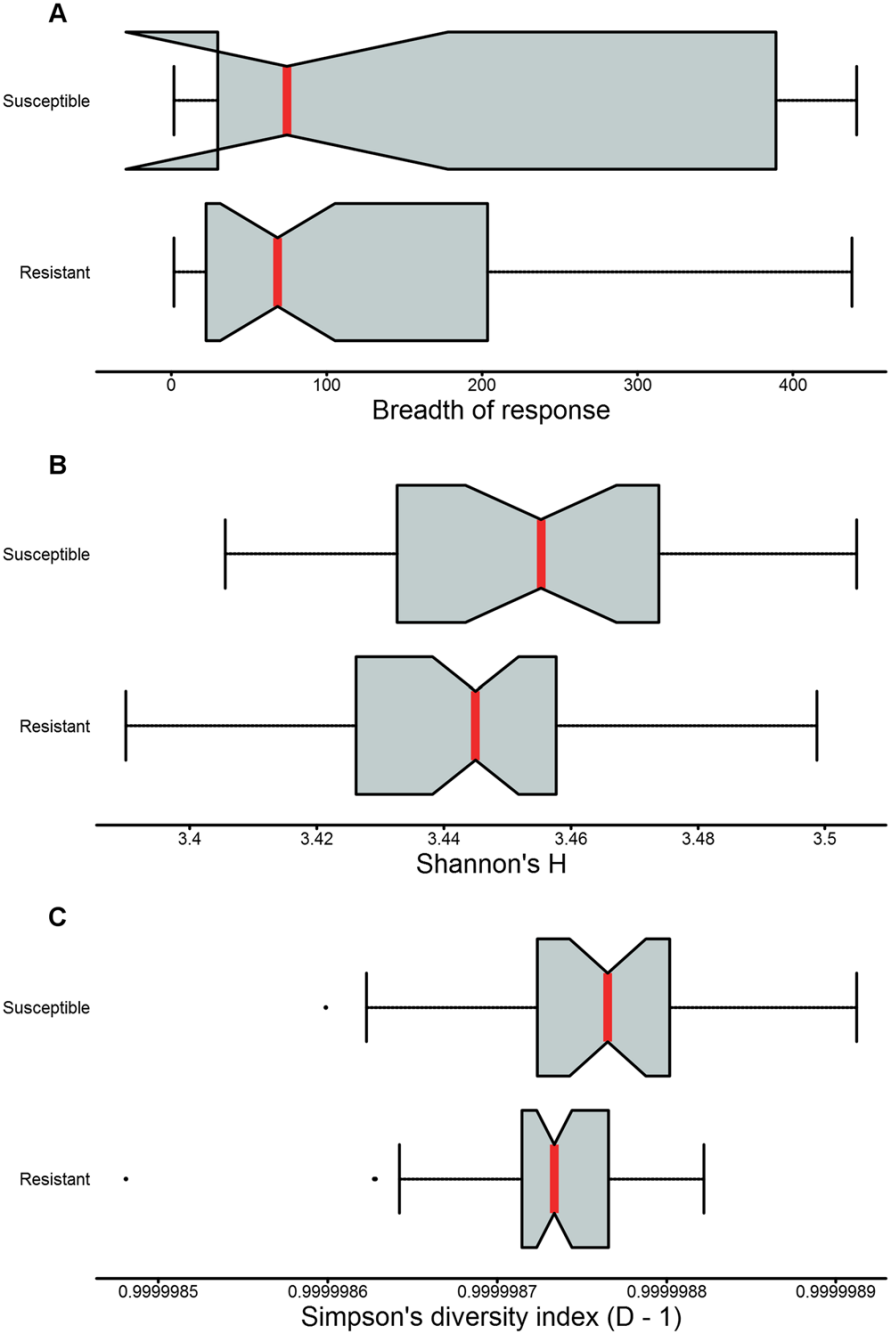
Breadth and diversity of responses in resistant and susceptible individuals. Notched boxplots of breadth/diversity of responses (x-axis) in resistant and susceptible individuals (y-axis). (**A**) Breadth measured as the number positive responses within individuals (p = 0.088). Diversity measured using (**B**) Shannon’s diversity index (p = 0.024) and (**C**) Simpson’s diversity index (p = 0.080). Median (red lines) and notches were calculated as the median +/− 1.57 × IQR/sqrt of n, where IQR is the interquartile range and n is the number of samples. The IQR times 1.5 was added to the 75th percentile and subtracted from the 25th percentile to determine the whiskers. Dots are outliers.

The complete profile of antibody responses in resistant and susceptible individuals was used to investigate differences in diversity or evenness of anti-Ct responses. Breadth, defined as the number of antigens recognised by an individual, was higher in susceptible individuals but this was not significant (p = 0.088) (Fig. 1A). Diversity indices, which incorporate breadth and the relative strength of responses against an antigen, were higher in susceptible individuals. This reached significance for Shannon’s (p = 0.024) but not Simpson’s diversity indices (p = 0.080) (Fig. 1B and C). Together these indicate broader, less focussed antibody responses in susceptible individuals.

### Individual antibody responses were associated with increased susceptibility

Association between responses to individual antigens and infection frequency and duration was determined using a generalised linear model adjusting for other major risk factors (age, gender and village of residence). Forty-two antigens were identified as targets of differential antibody responses between resistant and susceptible individuals (p ≤ 0.05) (Table 2). Higher responses to each of these antigens was associated with susceptibility to infection.

**Table 2:** Forty-two differentially recognised antigens between resistant and susceptible individuals.

Antigen	p-value	p*	t	SE(t)	OR*	95% CI*	AUC
**CT545**	0.001	< 0.001	8.38	2.63	9.05	2.57–39.71	0.78
**CT118**	0.003	0.002	6.40	2.18	8.18	2.19–37.48	0.78
**CT123**	0.004	0.001	6.43	2.21	8.7	2.23–43.16	0.76
**CT541**	0.006	0.004	6.09	2.21	6.74	1.85–28.71	0.78
**CT664**	0.009	0.008	5.76	2.20	6.36	1.71–28.27	0.80
**CT119**	0.009	0.007	5.19	1.98	4.35	1.51–14.00	0.76
**CT381**	0.010	0.009	5.03	1.96	4.56	1.48–15.49	0.76
**CT584**	0.012	0.010	6.90	2.75	6.3	1.62–29.64	0.76
**CT284**	0.012	0.009	6.34	2.53	4.83	1.51–18.22	0.74
**CT502**	0.014	0.012	6.46	2.62	5	1.50–19.93	0.80
**CT051**	0.017	0.015	5.06	2.11	5.44	1.47–24.26	0.74
**CT592**	0.019	0.018	4.14	1.77	3.6	1.28–11.23	0.75
**CT073**	0.020	0.017	6.01	2.58	6.91	1.47–39.52	0.77
**CT078**	0.020	0.018	6.07	2.61	4.88	1.38–20.58	0.74
**CT023**	0.022	0.019	6.60	2.88	7.61	1.56–51.76	0.77
**CT223**	0.022	0.020	5.93	2.59	4.11	1.31–15.14	0.76
**CT795**	0.024	0.018	3.91	1.73	3.67	1.23–11.93	0.76
**CT875**	0.028	0.030	5.14	2.34	4.21	1.22–16.47	0.73
**CT181**	0.030	0.024	4.32	1.99	4.05	1.20–15.42	0.75
**CT694**	0.031	0.031	3.91	1.81	3.68	1.17–12.78	0.74
**CT021**	0.034	0.033	5.45	2.57	4.72	1.19–21.77	0.77
**CT813**	0.035	0.033	3.50	1.66	3.13	1.12–9.57	0.75
**CT841**	0.036	0.035	3.77	1.80	3.15	1.12–9.78	0.74
**CT017**	0.036	0.039	3.77	1.80	4.37	1.16–18.92	0.75
**CT142**	0.036	0.037	4.24	2.03	3.41	1.12–11.48	0.74
**CT764**	0.037	0.034	5.74	2.75	4.5	1.18–20.92	0.78
**CT728**	0.039	0.040	4.86	2.35	3.97	1.14–16.23	0.78
**CT228**	0.039	0.037	5.54	2.69	4.34	1.16–19.64	0.74
**CT703**	0.040	0.042	4.82	2.34	5.76	1.20–34.90	0.74
**CT494**	0.040	0.037	5.63	2.74	5.62	1.19–32.96	0.76
**CT106**	0.041	0.039	5.87	2.87	3.45	1.11–12.30	0.76
**CT097**	0.041	0.040	5.50	2.70	3.6	1.10–13.26	0.75
**CT316**	0.043	0.049	6.00	2.96	3.66	1.10–14.02	0.75
**CT579**	0.044	0.041	3.73	1.85	3.55	1.09–13.26	0.77
**CT168**	0.044	0.044	6.32	3.14	5.37	1.19–32.48	0.76
**CT089**	0.044	0.045	4.54	2.26	3.34	1.07–11.61	0.75
**CT237**	0.045	0.043	5.79	2.88	4.53	1.13–22.39	0.75
**CT806**	0.046	0.046	4.89	2.45	3.49	1.08–12.84	0.76
**CT668**	0.048	0.049	5.68	2.87	3.45	1.07–12.91	0.77
**CT642**	0.048	0.047	5.80	2.93	3.39	1.08–12.52	0.76
**CT695**	0.049	0.053	3.73	1.90	3.44	1.06–12.84	0.75
**CT570**	0.050	0.049	5.92	3.02	4.29	1.08–20.48	0.76

Univariate associations were determined using a generalised linear model. Variables were resampled 10,000 times and remodelled to determine permuted p-values (P*). Odds ratios (OR) and confidence intervals (CI) were calculated for an increase of half the range per antigen, rather than one unit. The predictive value of the univariate generalised linear models was calculated and presented as area under the curve (AUC).

To examine when and how these antigens might be targeted by the host immune response during the Ct developmental cycle, their expression stage from Belland *et al*.^35^ and predicted localisation from three computational tools (loctree3, Cello and pSORTB) were compared between the 42 differentially recognised antigens and the 441 antigens (Supplementary Table 1). A χ^2^ test was used to quantify over-representation or under-representation of expression stage or predicted localisations in the differentially recognised antigens, compared to all 441 antigens. Very early (1 hour post infection [HPI]) and very late (24–36 HPI) expressed genes were significantly over-represented (p = 0.007). Secreted, inner membrane and periplasmic proteins were weakly over-represented in these antigens (p = 0.056). Experimentally defined localisation of these antigens showed mixed agreement with software predictions, a number of those known to be secreted or reside in the outer membrane were incorrectly classified as remaining inside the inclusion. There was less individually-determined data available for expression stage, although some antigens predicted to peak at 40 HPI have been shown since to peak 1–6 HPI. These disagreements, in classification of expression stage and localisation, strengthen the over-representation of antigens expressed early or late and localised to interact directly with the host.

### Genome-wide evidence of purifying and positive selection

In general, pathogen immunogenic proteins are under natural selection, due to their impact on pathogen survival and transmission. To validate identified immune targets and identify further targets, sequence data from 126 ocular Ct samples from the Bjiagos Islands, Guinea-Bissau was examined for evidence of departure from neutral selection. These samples were collected and sequenced as described elsewhere^33, 34^, details in Supplementary Methods. Currently, no Ct whole-genome sequences are available from the villages included in this study. To determine the relevance of evidence of selection in samples from the Bijagos Islands, they were compared with five historical isolates from The Gambia (Supplementary Figure 2). The Bijagos Islands samples were separated from each other by 1–1119 SNPs, the Gambian samples were separated by 161–1704 SNPs and the two populations of samples were separated by 487–1019 SNPs. As expected, based on previous Ct sequences, this suggests the geographically-distinct populations are close genetic relations, supporting the use of the Bijagos Islands samples in predicting genes under selection within those circulating in The Gambia. Therefore we tested for evidence of departure from neutrality using a number of tests including Tajima’s D, Fay and Wu’s H, and the integrated haplotype score.

Tajima’s D can distinguish between directional selection (positive or purifying selection [D ≤ −1.8]) and balancing selection (D ≥ 2.044), by comparing the levels of low and medium frequency alleles^36^. Fay and Wu’s H can distinguish between balancing/positive selection and balancing/purifying selection, by comparing the levels of low and high frequency alleles. Balancing (D ≥ 2.044 and 0.72 ≤ H ≤ −3.85), positive (D ≤ −1.8 and H ≤ −3.85) and purifying selection (D ≤ −1.8 and H ≥ 0.72) can be differentiated, by combining D- and H-values.

**Figure 2:**
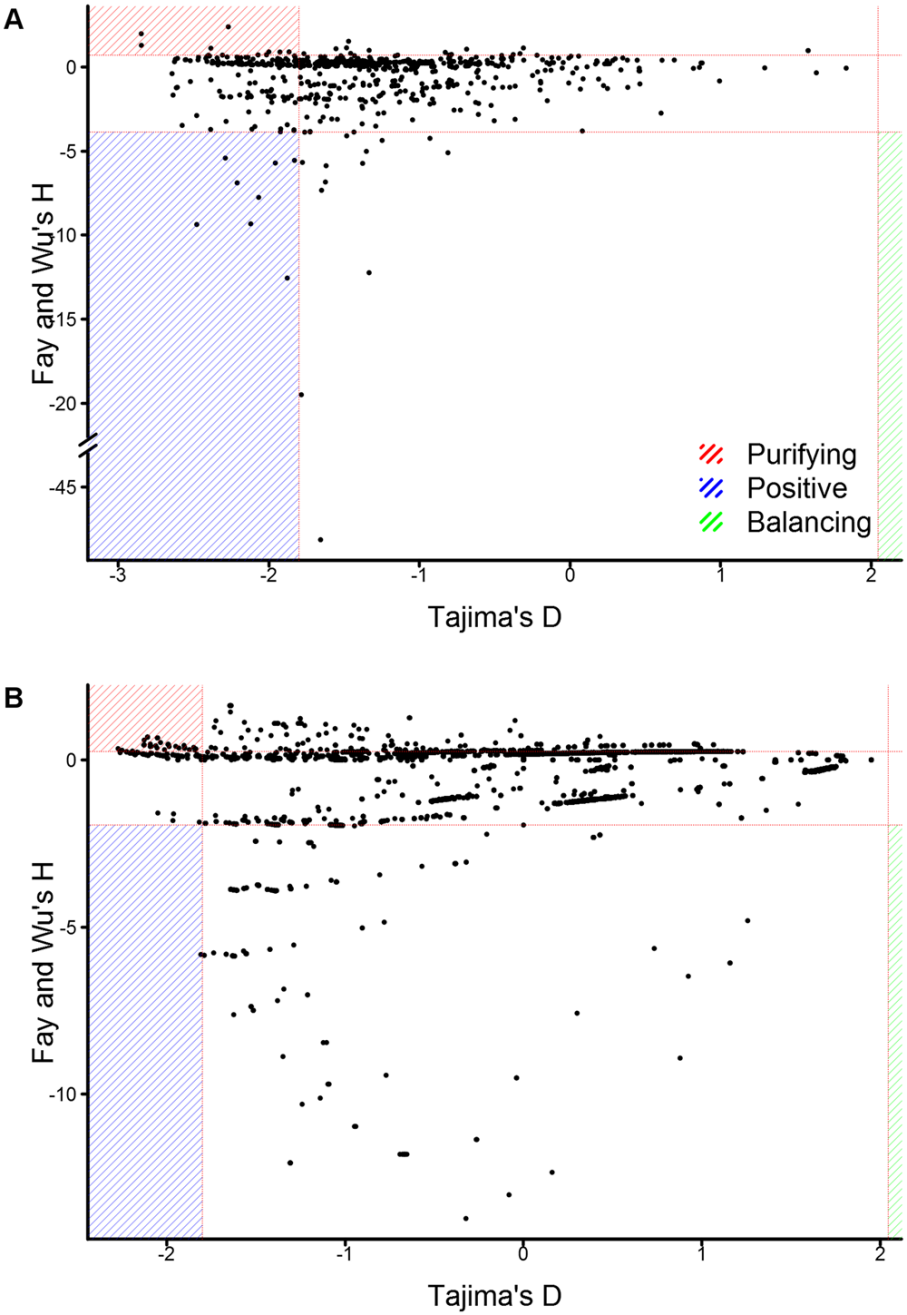
Correlation between Tajima’s D and Fay and Wu’s H. Genome-wide correlation of D and H values at (**A**) gene-level and (**B**) epitope-level (sliding windows of 42 bp). Values significantly different from zero are indicated for each measure (dashed red lines). Genes with evidence of positive (blue), purifying (red) and balancing selection (green) are highlighted.

Nineteen genes with evidence of selection by Tajima’s D were supported by Fay and Wu’s H. Ten of these genes had evidence of positive selection and 9 genes had evidence of purifying selection (Fig. 2A and Table 3 [‘Windows under selection’ = 0]). D- and H-values were then determined using a 42 base pair sliding window analysis, this window equates to the most frequent length of an antibody epitope (16 amino acids). Seventy-six windows across 12 genes had evidence of positive selection and 61 windows across 8 genes had evidence of purifying selection (Fig. 2B and Table 3 [‘Windows under selection’ >0]). Evidence of natural selection acting on/within these genes suggests they may influence Ct survival and transmission.

**Table 3:** Genes under selection identified by Tajima’s D and Fay and Wu’s H.

ID	Name	Number of SNPs	Fay and Wu’s H	Tajima’s D	Windows under selection	Type of Selection
**CT033**	*recD_1*	15	−5.41	−2.29	0	Positive
**CT046**	*hctB*	33	1.00	−1.33	2	Purifying
**CT082**		12	−9.32	−2.12	1	Positive
**CT105**		45	2.40	−2.27	13	Purifying
**CT147**		32	−12.23	−1.33	24	Positive
**CT159**		19	−3.23	−2.28	4	Positive
**CT223**	*IPAM*	15	0.91	−1.87	0	Purifying
**CT249**		5	−5.67	−1.78	1	Positive
**CT288**		12	−12.55	−1.88	0	Positive
**CT359**		8	−5.55	−1.83	1	Positive
**CT386**		7	−7.75	−2.07	0	Positive
**CT394**	*hrcA*	15	0.73	−2.01	0	Purifying
**CT414**	*pmpC*	41	−2.88	−1.52	16	Purifying
**CT442**	*crpA*	28	−0.86	−2.52	17	Purifying
**CT456**	*tarP*	32	−6.84	−1.62	8	Positive
**CT539**	*trxA*	4	−3.87	−1.76	2	Positive
**CT621**		16	0.90	−1.93	0	Purifying
**CT622**		47	−48.13	−1.66	4	Positive
**CT624**	*mviN*	17	0.91	−2.00	0	Purifying
**CT636**	*greA*	15	0.73	−2.07	0	Purifying
**CT641**	*ygeD*	42	1.13	−2.38	0	Purifying
**CT651**		51	0.32	−2.63	0	Purifying
**CT674**	*yscC*	51	1.30	−2.85	0	Purifying
**CT681**	*ompA*	25	−0.46	−2.20	13	Purifying
**CT686**	*sufD*	20	−19.50	−1.78	21	Positive
**CT688**	*parB*	7	−5.71	−1.96	3	Positive
**CT694**		14	−6.89	−2.21	6	Positive
**CT868**	*Dub1*	19	−9.36	−2.48	0	Positive
**CT872**	*pmpH*	68	1.99	−2.85	0	Purifying

Number of SNPs per gene and the gene-level Tajima’s D and Fay and Wu’s H values are indicated. The number of sliding windows with significant evidence of selection by both Fay and Wu’s H and Tajima’s D are indicated (‘Windows under selection’). Type of selection is indicated.

### Integrated haplotype scores identify three genomic regions under positive selection

Identification of positive selection by Tajima’s D and Fay and Wu’s H is most powerful when an allele is close to fixation (only one allele at a given site). A genome-wide scan was performed to calculate integrated haplotype scores (iHS) for SNPs, to identify genes and regions under positive selection that have not yet reached fixation.

**Figure 3:**
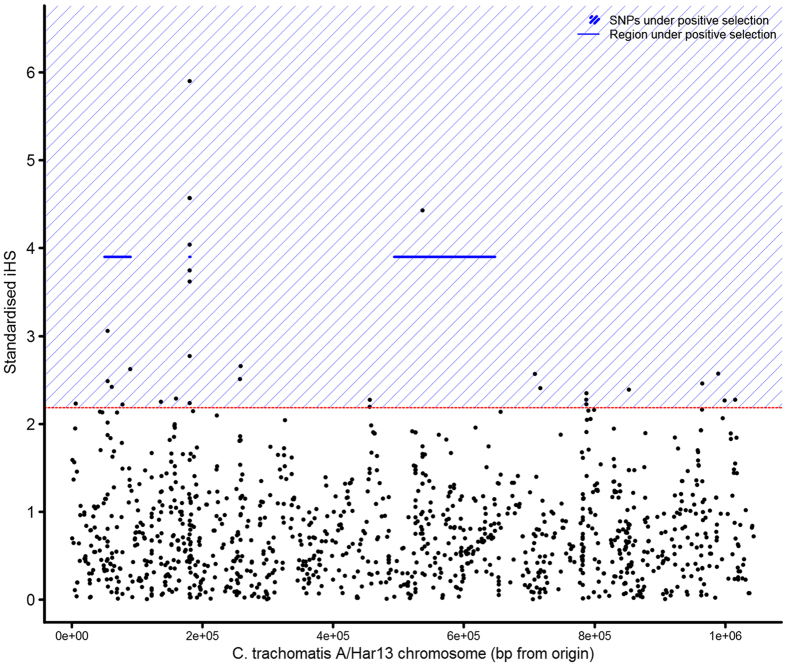
Evidence of positive selection using the integrated haplotype score. Genes in the top 1% of values (dashed red line) had the strongest evidence of positive selection. Regions (blue lines) and individual SNPs (blue shading) under positive selection are indicated.

The median iHS score was 0.66 (95% CI 0.03–2.18), 20 SNPs in the top 1% of the genome-wide distribution of iHS (Fig. 3). The top 1% of SNPs highlighted three loci, which showed evidence of recent positive selection; CT048-CT074, CT154-CT155 and CT456-CT625. These loci include a region covering *tarP* and *pmp* family members and a region within the Ct plasticity zone.

### Ct genes important for Ct survival and pathogenesis were the focus of natural selection

In this population of ocular Ct samples, 48 genes were identified with evidence of selection by either a combination of Tajima’s D and Fay and Wu’s H, at the gene or epitope level, or by iHS. Expression stage of these genes and localisation of the translated proteins was examined to discover common patterns in genes with evidence of selection (Supplementary Table 2). Secreted and outer membrane proteins were significantly over-represented in these targets (p = 0.004), as were genes with peak expression levels very early or very late in the developmental cycle (p = 0.0005). Expression at these pivotal stages of infection and extra-inclusion localisations suggests these genes are important factors in Ct survival and pathogenesis. Similar to the 42 antigens identified in this study, comparison of the array-determined expression stage and predicted localisation to more recent experiments supported these findings.

### Variable evidence of selection acting on genes associated with infection frequency and duration

**Figure 4:**
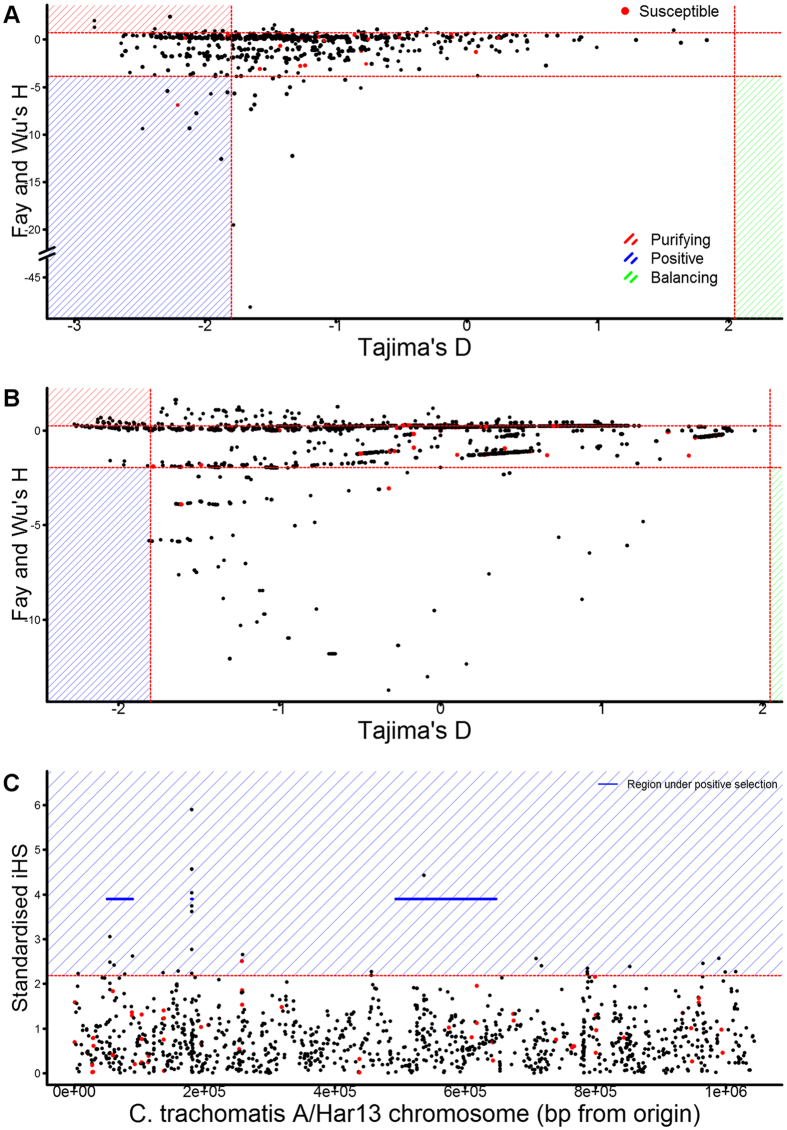
Evidence of selection in antibody targets associated with susceptibility to infection. Evidence of selection was determined at the gene-level (**A**) and the epitope-level (**B**) by Tajima’s D and Fay and Wu’s H. Evidence of positive selection on SNPs and larger genomic regions was independently determined by iHS (**C**). Association with susceptibility to infection is indicated (red). Thresholds for genes considered under selection, values significantly different from zero, is indicated for each measure (dashed red lines). Genes or SNPs (**C**) with evidence of positive (blue), purifying (red) and balancing selection (green) are highlighted.

Evidence of selection is a common marker of pathogen immunogenic proteins due to their interactions with the host and impact on pathogen survival and transmission. Therefore, we examined the 42 antigens associated with susceptibility to infection for evidence of selection, as one means of validation as important immune targets (Fig. 4 and Supplementary Table 3).

Four of 42 targets associated with susceptibility to infection had gene-level evidence of selection by Tajima’s D, supported by Fay and Wu’s H. CT694 and CT695, had evidence of positive selection at the epitope level supported by D and H values. CT545 and CT806 had evidence of purifying selection at the epitope level.

Ten genes were within regions under positive selection by iHS. One target, susceptibility-associated CT228, contained a SNP under positive selection by iHS.

The majority of the antigens associated with susceptibility had no evidence of selection in this population, suggesting they are evolving under neutral selection.

## Discussion

There is considerable evidence from animal models suggesting antibody responses are necessary for long-term protection and immunity from chlamydial infection^37–39^. In mice, the breadth of the antibody response is higher in strains that are more susceptible to chlamydial infection and associated pathology^40–42^. Furthermore, in non-human primates, partial immunity to ocular infection was consistent with development of a focussed antibody recall response^43^.

Previous work on the Gambian six-month longitudinal cohort utilised in this study focussed on systemic and local cell-mediated immune responses^44, 45^. To gain a more complete picture of the immune responses underlying differential outcomes, we screened serum samples against a Ct protein microarray to examine the relationship between individual serological immune responses and the acquisition and resolution of ocular Ct infection. Antibody responses were more focused in children who were able to resolve infection, while heightened responses to 42 antigens were associated with susceptibility to infection and longer durations of infection.

Tests for selection identified a number of genes and regions of the Ct genome under purifying and positive selection, using 126 ocular samples collected from the Bijagos Islands (Guinea-Bissau). These regions were focused in immunogenic proteins and those that interact directly with the host. This result was further strengthened by using experimentally derived expression stage and localisation of individually investigated genes, as opposed to array-based profiling of expression and bioinformatics prediction of localisation. Evidence for selection within the potential immune targets identified from the proteome array analysis was variable, 5/42 targets had significant evidence of selection.

Genes important in host-pathogen interactions are under natural selection. Most frequently, selection is observed in pathogen immune targets, where balancing and/or positive selection can aid immune evasion^46^. We found evidence of selection in Ct genes that code for known or putative immune targets or virulence factors, similar to previously described Ct genomics studies^24–27^. Genes coding for proteins involved in cell entry, cell exit and intracellular interactions via the inclusion membrane encompassed the majority of genes under selection. Additionally, 17 of 48 genes with evidence of selection have no known function, suggesting these genes may be important for Ct survival and transmission. Genome-wide scans for evidence of selection therefore have the ability to highlight important, previously uncharacterized genes.

Seventeen of the genes under selection are known to be immunogenic; this supports immune-recognition as a key driving factor of selection within this population of ocular Ct samples. However, there was limited evidence of balancing selection, a common mechanism of immune evasion employed by human pathogens^18, 21, 47, 48^. This method of immune evasion relies on cyclical presentation of alternative forms of immunodominant, primarily surface-exposed, antigens to the host immune system to avoid recognition and clearance.

Typically, in trachoma endemic communities where immunity develops slowly, Ct is able to reinfect individuals within the same households or village. The lack of protective antibodies, combined with the absence of balancing selection, suggests that Ct employs a different strategy for immune evasion. The results suggest two potential routes for immune evasion, a) blocking and/or invasion-enhancing antibodies against surface antigens or b) masking of protective humoral immune responses through heightened responses against a large number of immunogenic but non-protective antigens.

Blocking antibodies are induced by surface antigens of *P. falciparum* ^49^, *Candida albicans* and *Neisseria gonorrhoeae* and can inhibit protective responses^50, 51^. Antibody-dependent enhancement is a well-described process in viral infections^52, 53^, involving cross-linking of host-cell surface receptors by virus-antibody complexes leading to enhanced infectivity. Similar observations in *P. vivax* ^54^ suggest this mechanism may be more widely utilized by pathogens.

Through Ct-protein array screening of serum from children resistant or susceptible to frequent and/or prolonged Ct infection, we identified three susceptibility associated surface antigens CT017 (Ctad1), CT541 (MIP) and CT579. Antibodies generated against non-protective surface antigens provide a survival advantage for Ct and such mutations would be expected to be under purifying selection. There was no clear evidence of selection in these antigens. However, three of four outer membrane proteins (*ompA* [MOMP]﻿, *pmpC* and *pmpH*) with strong evidence of selection were under purifying selection, suggesting these antigens are targets of blocking and/or invasion-enhancing antibodies. In support of this, MOMP can induce blocking^55^ and invasion-enhancing antibodies^14, 56^ *in vitro* and in mouse models.

The majority of the 42 susceptibility associated antigens were not localised to the EB surface, therefore their role in Ct infection and disease cannot be explained by a hypothesis that solely involves non-protective surface antigens. The breadth of the antibody response shows that a high number of Ct antigens are accessible to the host immune system, likely through release of Ct proteins as a result of inclusion/host cell lysis or recently described chlamydial extrusions^57, 58^. Antibody responses against the majority of Ct proteins are not protective, leading to a hypothesis that heightened responses to a large number of immunogenic but not protective antigens masks protective humoral immune responses.

A diverse antibody profile would provide an advantage for the bacteria therefore targeted genes would be expected to be under purifying selection, however the number of antigens targeted and their inherently random nature implies no one gene would show strong evidence of selection. In support of evasion by decoy, 37/42 of the antigens identified as susceptibility-associated had no strong evidence of selection. Of the five under selection, two had evidence of purifying selection (CT545 and CT806). The remaining three were under positive selection (CT228, CT694 and CT695), however they are known virulence factors^59–63^, therefore immune evasion may not be the only driving force of selection.

Ideally, samples from the Gambian villages utilised in this study would have been screened for evidence of selection, however whole-genome sequences are currently unavailable. The phylogeny of the Bijagos Islands samples and the previously published Gambian samples, suggests they are closely related. This relatedness and the similar community-level endemicity of active trachoma (Gambian cohort 21.5% and Bijagos Islands 22% [one to nine year olds]) and ocular Ct infection (Gambian cohort 9.9% and Bijagos Islands 25% [one to nine year olds]), support the assertion that the genes under selection may be shared between these geographically-separated Ct populations. Additionally, evidence of diversity within the Bijagos Islands samples and historical Gambian samples, suggests observed differences in infection frequency and duration between villages are not due to the presence of a single dominant Ct clone.

These data demonstrate the value of genomic information in the identification of immune targets and virulence factors, particularly in combination with proteome-wide antibody responses. Less focussed antibody responses in susceptible individuals and evidence of purifying selection in Ct surface antigens, support an evasion strategy in which Ct presents a number of non-protective, irrelevant antigens to the immune system to block or misdirect protective responses. These results will allow targeted development of vaccine candidates and may explain the observed success of single and multi-antigen vaccines against Ct, as they would promote a focussed, protective humoral immune response. In summary, our data support the importance of antibody responses in human immunity to Ct infection.

## Methods

### Ethics statement

The Gambian longitudinal cohort was conducted in accordance with the Declaration of Helsinki. The study and its procedures were approved by the Gambia Government/Medical Research Council The Gambia Unit Joint Ethics Committee and by the Ethics Committee of the London School of Hygiene and Tropical Medicine (LSHTM). Verbal consent was obtained from community leaders. Written informed consent was obtained from all study participants’ guardians on their behalf. A signature or thumbprint is considered an appropriate record of consent in this setting by the above ethical bodies.

The Bissau-Guinean cross-sectional survey was conducted in accordance with the Declaration of Helsinki. The study and its procedures were approved by the Comitê Nacional de Ética e Saúde (Guinea-Bissau) and the LSHTM Ethics Committee. Verbal consent was obtained from community leaders. Written informed consent was obtained from all study participants or their guardians on their behalf if participants were children. A signature or thumbprint is considered an appropriate record of consent in this setting by the above ethical bodies.

### Clinical cohort study and participants

A six-month longitudinal study was previously conducted and recruited 345 children, aged four to fifteen years old, from nine villages in The Gambia^11^. Villages were selected following initial trachoma rapid assessment screening that found clinical signs of active trachoma in 20% of school age children. At baseline and at each fortnightly visit for six-months, children were examined for clinical signs of active trachoma. Trachoma was graded according to the simplified WHO grading system^64^. Two conjunctival swabs were collected in duplicate to test for Ct infection and tear fluid was collected for mucosal cytokine and antibody assays. Venous blood samples were collected at baseline and cessation of the study, 186 samples were collected. Villages 3 and 9 declined to give venous blood samples and could not be included in this study.

### Chlamydia trachomatis antigen microarrays

Ninety sera from baseline and 33 sera from the end of the study were screened against a previously published protein microarray covering 894 genomic open reading frames (ORFs) from serovar D Ct^32^. Briefly, 894 ORFs were cloned into the pXT7 expression vector, proteins were expressed and printed on glass slides. After blocking, the arrays were probed with diluted sera, bound antibody was detected using a biotin-conjugated anti-human antibody followed by a streptavidin-conjugated secondary antibody.

### Proteome microarray normalisation, filtering and clustering

The raw signal intensity data (Supplementary Table 4) from the protein micro-array was transformed by inverse hyperbolic sine transformation and normalised by mean-centring. To filter out infrequently recognised antigens, post-normalisation, the global median of the data was calculated and individual antigens whose median was lower than the global median were excluded from further analysis.

Different methods to identify positive-negative breakpoints in the distribution of the data were tested. Silhouette width was used to quantify the best method. Cluster separation per antigen was derived from the average silhouette width. To classify positive responses, two clusters were identified using the method that had the highest average silhouette width for each antigen.

### Diversity metrics

Breadth of response was defined as the number of antigens to which each individual had a positive response. Diversity was calculated using Shannon’s entropy (H) and Simpson’s diversity index (D).

### Statistical analyses

Intensity of responses was compared using a generalised linear model (glm) and the number of positive responses using Fisher’s exact test. For the glm, 10,000 permutations of the outcome variable were performed to generate an adjusted p-value. Adjusted odds ratios (OD*) and adjusted confidence intervals (CI*) were calculated as the unadjusted OR/CI exponentiated by half the range for a given antigen.

### Chlamydia trachomatis population genetic metrics

As part of a cross-sectional population-based survey in trachoma-endemic communities on the Bijagós Archipelago of Guinea-Bissau, upper tarsal conjunctival swabs were taken^33^. Whole-genome sequence data (Supplementary Table 5) was obtained from 126 Ct-positive swabs using SureSelect enrichment and Illumina paired-end DNA sequencing technology and assembled using the A/Har13 as reference genome. Aligned multi-fasta files for each gene were used as input for Variscan-2.0.3^65^ to calculate Tajima’s D and Fay and Wu’s H. Integrated haplotype scores (iHS) were calculated using the rehh package in R. Missing base-calls were imputed using a genetic-distance based method.

## Electronic supplementary material

Supplementary Information
