# Supplementary material for: Genome-wide profiling of humoral immunity and pathogen genes under selection identifies immune evasion tactics of Chlamydia trachomatis during ocular infection
Source: Sci Rep. 2017 Aug 29;7:9634. doi: 10.1038/s41598-017-09193-2 (PMC5575166; doi:10.1038/s41598-017-09193-2)
Supplement: Supplementary file 1 — Supplementary Information [file 41598_2017_9193_MOESM1_ESM.pdf]

Genome-wide profiling of humoral immunity and pathogen genes under selection identifies immune evasion tactics of *Chlamydia trachomatis* during ocular infection

Harry Pickering[1], Andy Teng[2], Nkoyo Faal[3], Hassan Joof[3], Pateh Makalo[3], Eunice Cassama[4], Meno Nabicassa[4], Anna R. Last[1], Sarah E. Burr[1,3], Sarah L. Rowland-Jones[3], Nicholas R. Thomson[5,6], Chrissy h. Roberts[1], David C. W. Mabey[1], Robin L. Bailey[1], Richard D. Hayward[7], Luis M. de la Maza[8], Martin J. Holland[1, 3].

[1]Clinical Research Department, Faculty of Infectious and Tropical Diseases, London School of Hygiene and Tropical Medicine, London WC1E 7HT, [2]ImmPORT Therapeutics, Inc./Antigen Discovery Inc., 1 Technology Dr., Suite E309, Irvine, CA 92618, United States, [3]Disease Control and Elimination Theme, Medical Research Council The Gambia Unit, Fajara, Banjul, The Gambia, [4]Programa Nacional de Saúde de Visão, Ministério de Saúde Publica, Bissau, Guiné-Bissau, [5]Department of Pathogen Molecular Biology, London School of Hygiene and Tropical Medicine, London WC1E 7HT, [6]Pathogen Genomics, Wellcome Trust Sanger Institute, Wellcome Trust Genome Campus, Hinxton, United Kingdom, [7]Institute of Structural and Molecular Biology, Birkbeck & University College London, Malet Street, London, WC1E 7HX, [8]Department of Pathology and Laboratory Medicine, Medical Sciences I, Room D440, University of California, Irvine, Irvine, CA 92697-4800, United States.

Corresponding author: harry.pickering@lshtm.ac.uk (HP)

Supplementary Figures

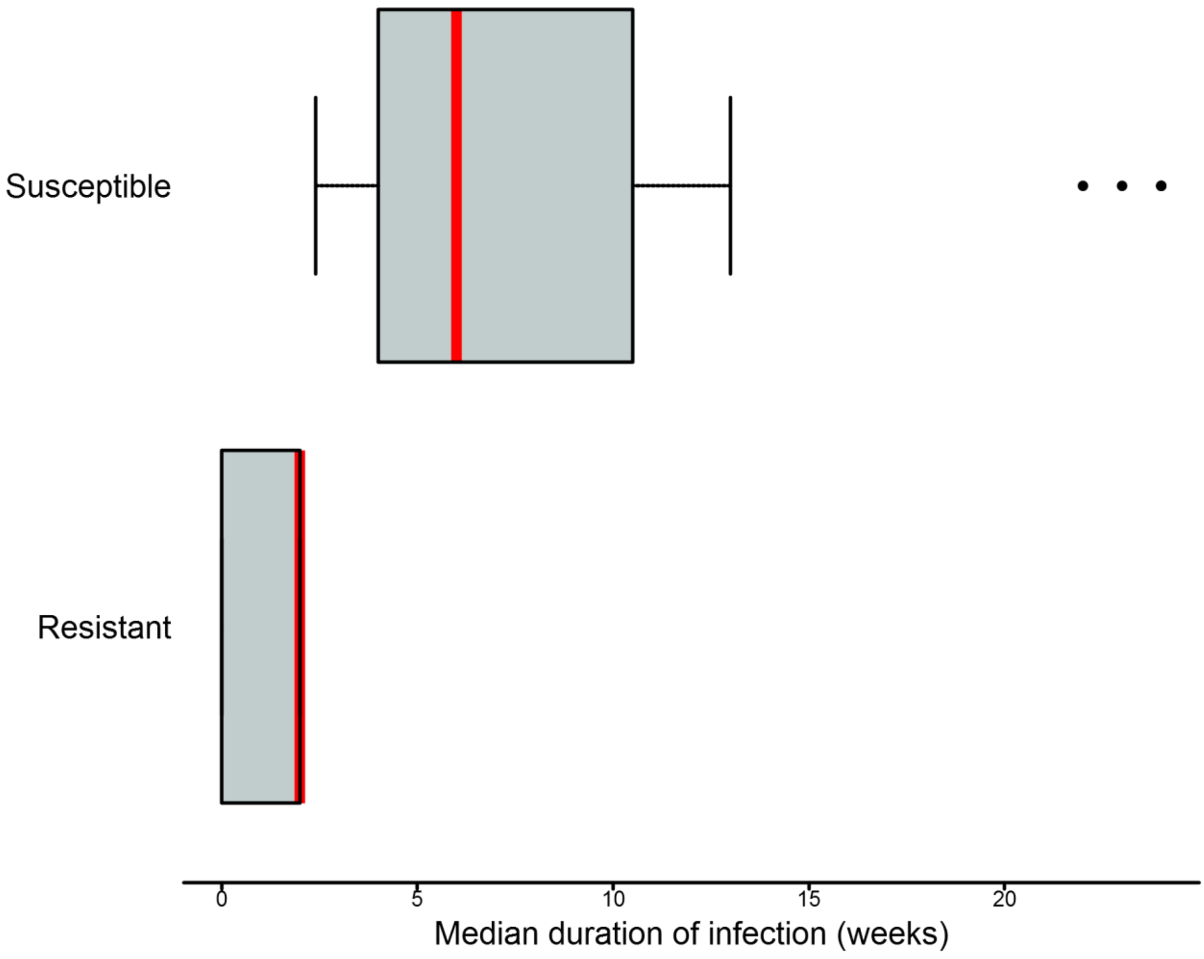

Supplementary Figure 1: Median duration of infection in resistant and susceptible individuals.

The median duration of infection in the cohort was 2 weeks. Individuals were dichotomised around the median according to their median duration of infection (2 weeks) into resistant (≤ 2 weeks) and susceptible (> 2 weeks). Red lines indicate the median. The whiskers were

calculated by adding 1.5 x IQR to the 75th percentile and subtracting 1.5 x IQR from the 25th percentile. Dots are outliers.

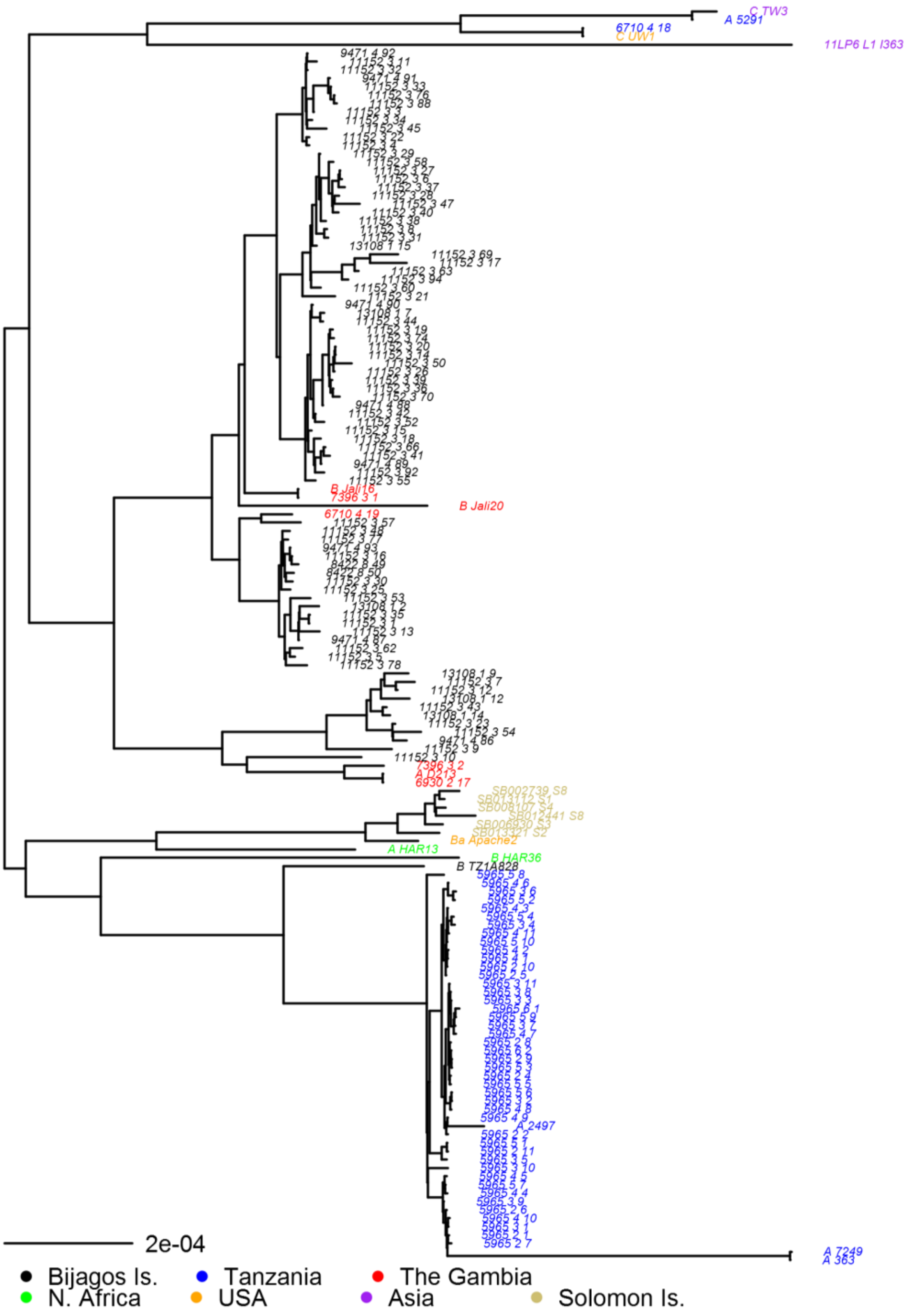

Supplementary Figure 2: Maximum likelihood reconstruction of the whole-genome phylogeny of 150 ocular Ct sequences.

Maximum likelihood reconstruction of the whole-genome phylogeny of 150 ocular Ct sequences from Asia (purple), Bijagós Islands (black), North Africa (green), Solomon Islands (yellow), Tanzania (blue), The Gambia (red) and USA (orange). Ct sequences were mapped to Ct A/HAR-13 using SAMtools80. Phylogenies were computed with RAxML from a variable sites alignment using a GTR+gamma model and are midpoint rooted. The scale bar indicates evolutionary distance.

Supplementary Tables

Supplementary Table 1: Predicted and experimentally determined expression stage and localisation of susceptibility-associated antigens.

ID	PREDICTED LOCALISATION	PEAK EXPRESSION (HPI)	LOCALISATION (BY MICROSCOPY OR IMMUNOBLOTTING	EXPRESSION (BY RT-qPCR OR MICROSCOPY [HPI])
**CT_017**	Cytoplasmic	40	Outer membrane[1]	1[1]
**CT_021**	Cytoplasmic	40		
**CT_023**	Secreted	40		
**CT_051**	Cytoplasmic	40	Inclusion lumen[2]	
**CT_073**	Secreted	24		
**CT_078**	Secreted	16		
**CT_089**	Cytoplasmic	40	Secreted[3]	
**CT_097**	Secreted	24		
**CT_106**	Cytoplasmic	24		
**CT_118**	Cytoplasmic	3-8	Inclusion membrane[4]	12[4]
**CT_119**	Secreted	24	Inclusion membrane[4]	20[4]

**CT_123**	Inner membrane	24		
**CT_142**	Cytoplasmic	24	Inclusion lumen[5]	
**CT_168**	Secreted	40		
**CT_181**	Cytoplasmic	24		
**CT_223**	Secreted	40	Inclusion membrane[6]	20[6]
**CT_228**	Inner membrane	3-8	Inclusion membrane[7]	6[7]
**CT_237**	Inner membrane	40		
**CT_284**	Cytoplasmic	40		
**CT_316**	Cytoplasmic	24		
**CT_381**	Cytoplasmic	40		
**CT_494**	Secreted	40		
**CT_502**	Inner membrane	40		
**CT_541**	Cytoplasmic	40	Outer membrane[8]	
**CT_545**	Periplasmic	40		
**CT_570**	Cytoplasmic	40		
**CT_579**	Inner membrane	40	Outer membrane[9]	
**CT_584**	Secreted	40	Secreted[10]	
**CT_592**	Cytoplasmic	40		
**CT_642**	Periplasmic	16		
**CT_664**	Inner membrane	40		
**CT_668**	Cytoplasmic	40	Secreted[11]	
**CT_694**	Secreted	40	Secreted[12]	6[12]
**CT_695**	NA	24	Secreted[10]	

**CT_703**	Cytoplasmic	24		
**CT_728**	Cytoplasmic	24	Cytoplasmic[13]	12[13]
**CT_764**	Inner membrane	24		
**CT_795**	Inner membrane	16	Secreted[14]	
**CT_806**	Inner membrane	40		
**CT_813**	Periplasmic	16	Inclusion membrane[4]	24[4]
**CT_841**	Cytoplasmic	40		
**CT_875**	Inner membrane	40	Secreted[15]	2[15]

Predicted localisations were defined using LocTree, Cello and psortB. Peak expression was defined from Belland *et al*[16]. Experimental localisation and RT-qPCR/microscopic expression data were determined using a PubMed literature search with three terms; “CT_xxx”, “CTA_xxxx” and “Chlamydia trachomatis”.

Supplementary Table 2: Predicted and experimentally determined expression stage and, localisation, and experimentally determined immunogenicity and function of genes with evidence of selection.

**ID**	**PREDICTED LOCALISATION**	**PEAK EXPRESSION (HPI)**	**IMMUNOGENIC**	**FUNCTION**	**LOCALISATION (BY MICROSCOPY OR IMMUNOBLOTTING**	**EXPRESSION (BY RT-qPCR OR MICROSCOPY [HPI])**
**CT_005**	Inner Membrane	16	No	Unknown	Inclusion membrane[4]	42[4]
**CT_033**	Cytoplasmic	16	No	Exodeoxyribonuclease		
**CT_046**	Extracellular	40	No	Histone-like protein		
**CT_048**	Cytoplasmic	24	No	Methyltransferase		
**CT_049**	Extracellular	40	No	Unknown	Inclusion lumen[2]	48[2]
**CT_050**	Extracellular	40	No	Unknown	Inclusion lumen[2]	48[2]
**CT_053**	Cytoplasmic	24	No	Unknown	Secreted[17]	20[17]
**CT_082**	Extracellular	24	Yes	Unknown	Secreted[10]	1[10]
**CT_105**	Extracellular	24	No	Unknown	Secreted[11]	2[11]
**CT_116**	Extracellular	3-8	Yes	Retromer recruitment	Inclusion membrane[18]	2[18]
**CT_141**	Cytoplasmic	40	No	Translocase		
**CT_147**	Cytoplasmic	3-8	Yes	Endosome interactions	Inclusion membrane[4]	2[4]
**CT_154**	Cytoplasmic	24	No	Phospholipase D		
**CT_157**	Cytoplasmic	40	Yes	Phospholipase D		
**CT_159**	Extracellular	40	No	Phospholipase D		
**CT_223**	Inner Membrane	40	Yes	Microtubule interactions	Inclusion membrane[6]	20[6]
**CT_228**	Inner Membrane	3-8	Yes	Cell exit regulation	Inclusion membrane[7]	6[7]
**CT_229**	Extracellular	3-8	Yes	Rab recruitment	Inclusion membrane[19]	2[19]

**CT_249**	Extracellular	16	No	Unknown	Inclusion membrane[4]	2[4]
**CT_288**	Inner Membrane	3-8	Yes	Unknown	Inclusion membrane[4]	43[4]
**CT_359**	Inner Membrane	40	Yes	Unknown		
**CT_386**	Cytoplasmic	40	No	Unknown		
**CT_394**	Cytoplasmic	40	No	Transcription repressor		
**CT_396**	Cytoplasmic	16	Yes	Chaperone	Outer membrane[9]	
**CT_414**	Extracellular	24	Yes	Adhesion	Outer membrane[20]	24[20]
**CT_442**	Outer Membrane	24	Yes	Unknown	Inclusion membrane[4]	42[4]
**CT_456**	Extracellular	40	Yes	Cell entry/actin reorganisation	Outer membrane[21]	1[21]
**CT_539**	Cytoplasmic	24	No	Thioredoxin	Periplasmic[22]	
**CT_621**	Extracellular	40	Yes	Unknown	Secreted[23]	16[23]
**CT_622**	Cytoplasmic	24	No	Unknown	Secreted[24]	6[24]
**CT_624**	Inner Membrane	40	No	Lipid II flippase		
**CT_626**	Cytoplasmic	40	No	Protein synthesis		
**CT_636**	Cytoplasmic	24	No	Transcription		
**CT_641**	Inner Membrane	40	No	Membrane efflux		
**CT_651**	Cytoplasmic	40	No	Unknown		
**CT_674**	Outer Membrane	40	No	Type-3 secretion		
**CT_680**	Cytoplasmic	40	No	Protein synthesis		
**CT_681**	Outer Membrane	24	Yes	Adhesion	Outer membrane[25]	24[4]
**CT_683**	Cytoplasmic	40	No	Unknown		
**CT_686**	Cytoplasmic	40	No	Electron transfer		
**CT_688**	Cytoplasmic	40	No	Cell division		
**CT_694**	Cytoplasmic	40	Yes	Actin reorganisation	Secreted[12]	6[12]
**CT_818**	Inner Membrane	40	No	Tyrosine transport		
**CT_837**	Cytoplasmic	40	No	Unknown		
**CT_845**	Cytoplasmic	24	No	Unknown		

**CT_859**	Cytoplasmic	40	No	Cellular metabolism		
**CT_868**	Extracellular	40	No	Deubiquitination	Secreted[26]	48[26]
**CT_872**	Extracellular	40	Yes	Adhesion	Outer membrane[9]	

Predicted localisations were defined using LocTree, Cello and psortB. Peak expression was defined from Belland *et al*[16]. Experimental localisation and RT-qPCR/microscopic expression data were determined using a PubMed literature search with three terms; “CT_xxx”, “CTA_xxxx” and “Chlamydia trachomatis”. Immunogenicity and function were manually assigned from PubMed literature searches.

Supplementary Table 3: Evidence of selection in immune targets from screening of the Ct-proteome micro-array.

Genes are ordered by position in the Ct D/UW3 genome. The number of sliding windows with significant evidence of selection in the respective gene is indicated by ‘Fay and Wu’s H SW’ and ‘Tajima’s D SW’. Genes which fall within the three regions identified as under positive selection using the top 1 % of SNPs are indicated by ‘iHS window’. Genes with significant evidence of selection are highlighted (light red).

ID	NUMBER OF SNPS	THETA	TAJIMA’S D	TAJIMA’S D SW	FAY AND WU’S H	FAY AND WU’S H SW	IHS WINDOW
**CT017**	8	0.001	-1.78		0.12		N
**CT021**	8	0.002	-1.16		0.37		N
**CT023**	8	0.001	-0.61		0.03	14	N
**CT051**	11	0.001	-1.43		0.30		Y
**CT073**	7	0.001	-1.24		-0.66	14	Y
**CT078**	4	0.001	-1.13		-2.72		N
**CT089**	7	0.001	-1.27		0.29		N
**CT097**	5	0.001	-0.86		-2.77	7	N
**CT106**	5	0.001	-1.74		0.49		N

**CT118**	4	0.002	-0.12	0.18	19	N
**CT119**	1	< 0.001	-1.04	0.57		N
**CT123**	2	0.001	-1.31	0.02		N
**CT142**	4	0.001	-1.77	0.06		N
**CT168**	4	0.002	-1.73	0.09		N
**CT181**	3	0.001	-1.61	0.10		N
**CT223**	3	0.001	-0.81	0.07		N
**CT228**	8	0.003	0.069	-1.18		N
**CT237**	3	0.001	-1.62	-1.30		N
**CT284**	6	0.001	-1.58	0.07	6	N
**CT316**	1	< 0.001	-1.03	-3.09		N
**CT381**	0	0.000	NA	0.02		N
**CT494**	5	0.001	-1.86	0.00		Y
**CT502**	1	< 0.001	-1.03	0.13		Y
**CT541**	2	0.001	-0.11	0.02		Y
**CT545**	9	< 0.001	-1.82	0.27	14	Y

**CT570**	6	0.001	-0.75	14	0.44		Y
**CT579**	8	0.001	-2.15		0.03		Y
**CT584**	0	0.000	NA		0.18		Y
**CT592**	11	0.001	-1.83		0.00		Y
**CT642**	20	0.005	-2.47		0.59		N
**CT664**	7	0.001	-1.41		0.59		N
**CT668**	6	0.002	-1.58		0.52		N
**CT694**	14	0.003	-2.21		0.31	13	N
**CT695**	7	0.001	-0.77		-6.89	14	N
**CT703**	3	< 0.001	-1.62		-2.56		N
**CT728**	2	0.001	-1.38		0.48		N
**CT764**	2	< 0.001	-1.38		0.42		N
**CT795**	1	< 0.001	0.24		0.04		N
**CT806**	12	0.001	-1.83		0.20	5	N
**CT813**	2	< 0.001	-0.52		0.67		N
**CT841**	7	0.001	-1.10		0.22		N

**CT875**	9	0.001	-1.27	-0.09	N

Supplementary Table 4: Microarray raw signal intensity data (provided as individual file).

Supplementary Table 5: EBI-ENA accession numbers for raw read data from sequenced Ct isolates.

Supplementary Methods

Clinical cohort study and participants

In 2002 a rapid assessment survey of adults and children was carried out in the Western and North Bank Regions of The Gambia and villages with greater than 20 % prevalence of active trachoma (TF and/or TI) were selected [27,28]. The study was designed to measure time to resolution and time to acquisition of infection and disease. Based on census data and previous studies on the household pattern of disease we expected 100 diseased subjects and 150 non-diseased subjects at baseline, with > 80 % power to detect an adverse factor that doubles median infection/disease time in survival analysis. A further three villages were included to increase the power of study as the prevalence of active trachoma in the target population (children aged four to fifteen years old) was lower than expected. The joint Gambian Government-Medical Research Council Ethics Committee and the Ethics Committee of the London School of Hygiene & Tropical Medicine approved the design and procedures of this study.

School-age children in these villages were examined for the clinical signs of trachoma. A subset of 345 children between the ages of four and fifteen years old were recruited from households with a case of active trachoma and followed for a period of 28 weeks. At baseline and approximately fortnightly visits (ten to nineteen days), children were examined for signs of active trachoma. Two swabs were collected, one into a dry polypropylene tube and the other into RNAlater™. Tear fluid was collected from the right eye using a sponge-tipped eye spear (Merocel®, Xomed Surgical Products, Jacksonville, FL,

USA), inserted in the inferior conjunctival fornix and held there for approximately 30 seconds. Participants from one village withdrew consent en masse, after eight weeks. The completeness of follow-up was further influenced by individuals being absent from the village on visit days or travelling out of the area for the remainder of the study period [29]. An episode of infection was defined as a positive result from an in-house 16S rRNA PCR [30], clinical disease was defined according the WHO simplified grading system [31]. A subgroup of 135 participants consented to venipuncture at the beginning of the study and 105 at cessation of the study. Collectively, 130 serum samples from baseline and cessation of the study were available for testing.

For the classification of clinical categories and further analysis an episode of infection was defined as a Ct-positive result from an in-house 16S RNA RT-qPCR assay [30], an episode of disease was defined as presence of either follicular trachoma (TF) or inflammatory trachoma (TI) [31]. An episode was considered continuous if an individual's infection or disease status was consistent in consecutive visits, where data was missing between visits with inconsistent infection or disease status it was assumed status changed at the midpoint.

Chlamydia trachomatis antigen microarrays

Ct protein microarray chips were prepared as described previously [32] by Antigen Discovery (Irvine, CA) and screened using sera from 123 patients. Briefly 894 ORFs from the Ct D/UW3-CW genome were PCR amplified and *in vivo* cloned into the pXT7 expression vector which expresses proteins with an N-terminal His fragment and a C-terminal haemagglutinin sequence and T7 terminator. Ct-specific products were expressed from the plasmids using an *in vitro* transcription translation system (RTS 100 kit, Roche Diagnostics, West Sussex, UK) and printed on nitrocellulose coated glass slides (GraceBio, Bend, OR, USA) using an OmniGrid Accent microarray printer (Digilab, Marlborough, MA, USA).

Successful expression of the proteins was determined using antibodies against His (clone His-1; Sigma) and haemagglutinin (clone 3F10; Roche Diagnostics, West Sussex, UK).

Prior to testing sera was diluted 1/100 in blocking buffer (10 % *Escherichia coli* lysate [McLab, San Francisco, CA, USA] in protein array blocking buffer [Whatman, Piscataway, NJ, USA]) at room temperature for 30 minutes with agitation while the microarrays were rehydrated using protein array blocking buffer (Whatman, Piscataway, NJ, USA). The arrays were interrogated with sera at room temperature for 2 hours with agitation. After 3 washes with washing buffer (0.05 % Triton X-100 in phosphate-buffered saline (PBS) at pH 7.5) the microarrays were incubated with biotin-conjugated goat anti-human antibody (Jackson ImmunoResearch Laboratories, West Grove, PA, USA). After 3 washes the microarrays were incubated with streptavidin-conjugated Sensilight P3 (Columbia Biosciences, Columbia, MD, USA). The microarrays were scanned using a ScanArray Express HT microarray scanner (Perkin Elmer, Waltham, MA, USA) and the fluorescence signal was quantified and corrected for background noise using QuantArray software (Perkin Elmer, Waltham, MA, USA).

Proteome microarray normalisation, filtering and clustering

The raw signal intensity data from the microarray was transformed by inverse hyperbolic sine transformation and normalised by mean-centring, these techniques were determined as the most suitable 'normalisation' step using relevant rank deviation (RRD) [33]. Post-normalisation the global median of the data was calculated, individual antigens whose median was lower than the global median were excluded.

Several different methods were tested to identify positive-negative breakpoints in the distribution of the data. We tested extrinsic and intrinsic methods. Extrinsic methods tested were; mean of no DNA controls, global mean and global mean plus 2 standard deviations.

Intrinsic methods tested were; k-means clustering, k-medoids clustering, fuzzy c-means clustering, hierarchical clustering and mixture modelling. The intrinsic methods were tested allowing for 2 to10 clusters. The average silhouette width of each antigen was used to determine appropriateness of the cluster configuration. Silhouette ranges from -1 to +1 and is defined by equation 1.

$$\text{silhouette}(i) = \big(b(i) - a(i)\big)/max\{a(i), b(i)\} \qquad (1)$$

Where *i* is a data point, *a* is average dissimilarity with all other data points in its cluster, *b* is the lowest average dissimilarity to any other cluster of which *i* is not a member. If *i* is similar to other data in its cluster *a* will be low. If *i* is also dissimilar to data in the nearest cluster *b* will be high. In this case silhouette will tend towards +1. If *i* is not similar to its cluster *a* will be high. If *i* is similar to data in the nearest cluster *b* will be low. In this case silhouette will tend towards -1.

The mean of each silhouette per antigen resulted in the average silhouette width, which was a measure of the appropriateness of the cluster configuration. To determine positive responses, two clusters were identified and the method which had the highest average silhouette width for each antigen was identified. Data points clustered with the maximum OD/signal intensity point of each antigen were considered positive and the opposing cluster negative.

Diversity metrics

Ecological measures of diversity rely on species breadth/richness, the total number of species in a sample, and species diversity, which additionally incorporates the relative abundance of each species. In this analysis antigens were considered as species, abundance as the response to each antigen and the samples were either the complete data set or split into the

dichotomous outcome variables. These definitions are based on the assumption that responses on the array correlate with abundance of circulating antibodies in each sample. A normalised OD of 1 unit was interpreted as 1 arbitrary unit of circulating antibody. This means if a response to an antigen is twice the level in one sample compared with another, circulating antibodies are twice as abundant in that individual.

Breadth was defined as the number of antigens to which each individual made a positive response. For the remaining measures examining diversity existing methods were adapted to incorporate the continuous OD/signal intensity values. This was deemed more appropriate as an assumption of these methods was that individuals within a species are equivalent [34,35], in this analysis the species are antigens and positive responses within them are not equal.

We utilised two different measures of diversity to improve reliability of the results. Shannon's entropy (*H*) defined by equation 2, Simpson's index (*D*) defined by equation 3 and Hill numbers defined by equation 4 [35]. Higher values for all three indicate increased diversity and greater evenness. High values of *H* mean 1 unit of antibody in a sample could be targeted against any antigen because responses in the sample are even [36]. High values of *D* mean that 2 separate units of antibody from the array they are unlikely to be targeted against the same antigen due to evenness of the responses [36].

$$H = -\sum_{i=1}^{S} p_i \log p_i \qquad (2)$$

$$D = 1 - \sum_{i=1}^{S} {p_i}^2 \qquad (3)$$

Where S is number of antigens and p_i is the proportion of antibodies specific to each antigen. P_i is estimated as the amount of antibody specific to each antigen divided by the total amount of antibody present in each individual.

Chlamydia trachomatis population genetics metrics

Survey, clinical examination and sample collection methods have been described previously[37]. Briefly, we conducted a cross-sectional population-based survey in trachoma-endemic communities on the Bijagós Archipelago of Guinea Bissau. Conjunctival swabs were obtained from the left upper tarsal conjunctiva of each participant, DNA was extracted and Ct omcB (genomic) copies/swab quantified from the second conjunctival swab using droplet digital PCR (ddPCR) [38].

For 8 individuals, whole genome sequence (WGS) data was obtained following Ct isolation in cell culture. For the remaining individuals (118), WGS data were obtained directly from clinical samples. DNA baits spanning the length of the Ct genome were compiled by SureDesign and synthesized by SureSelectXT (Agilent Technologies, Santa Clara, CA, USA). Ct DNA extract from clinical samples was quantified and carrier human genomic DNA added to obtain a total of 3µg input for library preparation. DNA was sheared using a Covaris E210 acoustic focusing unit [39]. End-repair, non-templated addition of 3'–A adapter ligation, hybridisation, enrichment PCR and all post- reaction clean-up steps were performed according to the SureSelectXT Illumina Paired-End Sequencing Library protocol (V1.4.1 Sept 2012). All recommended quality control measures were performed between

steps. DNA was sequenced at the Wellcome Trust Sanger Institute using Illumina paired-end technology (Illumina GAII or HiSeq 2000). All 126 sequences passed standard FastQC quality control criteria [40]. Sequence data is available from the European Bioinformatics Institute (EBI) short read archive (Supplementary Table 3).

Alignment, assembly and filtering by individual genes

Raw fastq files were aligned and assembled using BWA SAMtools [41] with A/Har-13 as the reference genome. Variants were called and filtered using BCFtools [42] and VCFtools [43], with a minimum base quality score of 20 (99% accuracy) and a minimum read depth of 10.

Assembled sequences were combined and used as a database in the command-line version of Basic Local Alignment Search Tool (BLAST+)[44]. Individual gene sequences from A/Har-13 were used as queries to extract copies successfully sequenced in the isolates. Sequences with more than half missing calls were excluded. MUSCLE algorithm was used for gene alignments [45]. Alignments were inspected manually using SeaView [46] and visualisation were output using Geneious [47].

Allele frequency-based signatures of selection

Aligned multi-fasta files for each gene were used as input for Variscan-2.0.3 [48] to calculate Tajima's D, Fu and Li's D* and F* and Fay and Wu's H. RunMode 12 and RunMode 22 were used, sites with less than 50 sequences were not included. Sliding-window analyses were performed over windows of 42 nucleotides with jumps of three nucleotides. All three measures look at the number and frequency of mutations within a population to determine whether they occurred randomly under neutrality or were caused by a form of natural selection. They are based on different methods of estimating the genetic diversity (θ) in a population [49].

Tajima's *D* compares the average pairwise diversity (π), the average difference between a pair of sequences across all sites, and the number of segregating sites (κ), the number of sites within a population which are polymorphic [50]. Tajima's D is calculated from equation 7. For equation 5, *x* is the frequency of sequences *i* and *j*, *δ* is the number of nucleotide differences per site between them and *N* is the total number of sequences. For equation 6, *n* is the number of sequences and *i* is the number of times a given allele is present.

$$\theta_\pi = \sum x_i x_j \delta ij/N \quad (5)$$

$$\theta_\kappa = \frac{\kappa}{\sum_i^{n-1} 1/i} \quad (6)$$

$$D = \theta_\pi - \theta_\kappa \quad (7)$$

Positive selection increases the frequency of a few advantageous mutations, meaning most mutations are kept at a low frequency. In this situation the average difference between pairs of sequences is low but the number of segregating sites is relatively high, thus *D* is negative. Purifying selection reduces the frequency of deleterious mutations, meaning mutations occur but not become common. In this situation the average difference between pairs of sequences is low and the number of segregating sites is high, thus *D* is negative. Balancing selection maintains multiple mutations at intermediate frequencies, this results in increased polymorphism at these sites. In this situation the average difference between pairs of sequences is higher but the number of segregating sites remains stable, thus *D* is positive. A limitation of Tajima's D is the influence of population changes. Population expansion will increase the number of low frequency mutations making D negative, population bottlenecks will reduce the number of low frequency mutations making D less negative.

Fay and Wu's *H* can be used to determine which form of natural selection is occurring, or more accurately which is the dominant selection pressure. Fay and Wu's *H* utilises an outgroup sequence (A/Har-13) and focusses on the difference between intermediate and high frequency alleles rather than intermediate and low frequency alleles [51]. *H* is calculated from equation 9. Where *n* is the number of sequences, *i* is the number of times an allele occurs and ξ_i is the number of differences from the ancestral outgroup per allele.

$$\theta_\pi = 2/n(n-1)\sum_{i=1}^{n-1} i(n-i)\xi_i \qquad (8)$$

$$\theta_H = 2/n(n-1)\sum_{i=1}^{n-1} i^2\xi_i \qquad (9)$$

$$H = \theta_\pi - \theta_H \qquad (10)$$

H is heavily influenced by high frequency alleles because of the i^2 component in equation 9. Purifying selection keeps alleles from becoming common, therefore are few high frequency alleles and *H* is positive. Positive selection causes alleles to rise to fixation and thus increase in frequency, in these situations *H* is negative.

Haplotype-based signatures of selection

These metrics utilising allele frequencies were complemented by a haplotype based method, the integrated haplotype score (iHS). iHS identifies alleles at intermediate frequencies that are being driven towards fixation (positive selection) or under balancing selection [52]. Extended haplotype homozygosity (EHH) is a measure of distance calculated around SNPs on a given haplotype, if the haplotype is all the same EHH will be 1 and if it is all different EHH will be 0. Integrated haplotype homozygosity (iHH) is calculated as the decrease or

decay in EHH with genetic distance from a given SNP, both on the ancestral sequence and the derived sequence. In our studies the ancestral allele was defined as the allele in A/Har-13 and the derived allele was defined by variants in the Ct isolates from Guinea-Bissau. The unstandardised iHS is calculated from equation 13. Where iHHA and iHHD are the iHH from the ancestral and derived sequences respectively.

$$unstandardised\ iHS = \ln\left(\frac{iHHA}{iHHD}\right) \tag{13}$$

Unstandardised iHS is then standardised based on the genome-wide mean and variance of iHS for SNPs with the same allele frequency. If multiple SNPs with evidence of selection are within a defined distance of each other, EHH greater than 0.05, they can be combined to indicate windows under selection. Values significantly <1 indicate longer haplotypes on the derived sequence, indicative of a selective sweep driving an allele towards fixation before further mutations have arisen. Values significantly > 1 indicate longer haplotypes in the ancestral sequence, which is a sign of selection. Selection could now be favouring the ancestral allele or ancestral alleles around the favoured site could be hitchhiking. In our analysis we used the absolute iHS, classifying significantly positive values as indicators of selection.

Short read data from the 126 ocular Ct samples were mapped against Ct A/HAR-13 using SAMtools [17]. Non-polymorphic sites were removed. SNPs with a minor allele frequency (MAF) less than 0.05 and more than 25 % missing calls were excluded. Alleles were defined as ancestral (0) or derived (1) based on the ancestral isolate A/Har-13. iHS cannot be calculated over sites with missing base-calls, for this reason imputation was used to classify missing base-calls. A simple genetic distance-based imputation was used. Genome-wide pairwise nucleotide diversity was calculated for each pair of sequences. For each

missing base-call within a sequence, all sequences with base-calls at those sites were assigned a score based on the pairwise diversity between them and the sequence with a missing base-call. These scores were summed at each site for ancestral and derived base-calls respectively and divided by the number of respective base-calls. The state with the lower score was used to define the missing base-call. iHS was calculated using the R package rehh, Scores were standardised as described above after binning MAF into 40 discrete bins of size 0.025 [0.000-0.025, 0.025-0.05,..…,0.950-1.000].

Molecular and functional characterisation of target genes and proteins

Developmental cycle expression stage for each transcript was based on data and groupings from Belland et al[16], this grouping was manually assigned to data from Nicholson et al[53]. Localisation of expressed proteins was predicted using Cello[54], pSORTB[55] and loctree3[56], three of the top performing servers for bacterial proteins[56]. Predicted localisation was defined as the consensus from the 3 predictions. Immunogenicity and function were determined through a PubMed literature search with three terms; “CT_xxx”, “CTA_xxxx” and “Chlamydia trachomatis”. Similarly, expression stage and localisation predictions were validated through a PubMed literature search, for evidence of expression determined by RT-qPCR and localisation determined by microscopy or immunoblotting.

Supplementary References

1 Stallmann, S. & Hegemann, J. H. The Chlamydia trachomatis Ctad1 invasin exploits the human integrin beta1 receptor for host cell entry. *Cellular microbiology* **18**, 761-775 (2016).

2 Jorgensen, I. & Valdivia, R. H. Pmp-like proteins Pls1 and Pls2 are secreted into the lumen of the Chlamydia trachomatis inclusion. *Infection and immunity* **76**, 3940-3950 (2008).

3 Fields, K. A. & Hackstadt, T. Evidence for the secretion of Chlamydia trachomatis CopN by a type III secretion mechanism. *Molecular microbiology* **38**, 1048-1060 (2000).

4 Almeida, F. *et al.* Polymorphisms in inc proteins and differential expression of inc genes among Chlamydia trachomatis strains correlate with invasiveness and tropism of lymphogranuloma venereum isolates. *Journal of bacteriology* **194**, 6574-6585 (2012).

5 da Cunha, M., Pais, S. V., Bugalhao, J. N. & Mota, L. J. The Chlamydia trachomatis type III secretion substrates CT142, CT143, and CT144 are secreted into the lumen of the inclusion. *PloS one* **12**, e0178856 (2017).

6 Dumoux, M., Menny, A., Delacour, D. & Hayward, R. D. A Chlamydia effector recruits CEP170 to reprogram host microtubule organization. *J Cell Sci* **128**, 3420-3434 (2015).

7 Lutter, E. I., Barger, A. C., Nair, V. & Hackstadt, T. *Chlamydia trachomati*s inclusion membrane protein CT228 recruits elements of the myosin phosphatase pathway to regulate release mechanisms. *Cell reports* **3**, 1921-1931 (2013).

8 Lundemose, A. G. *et al.* Chlamydia trachomatis Mip-like protein. *Molecular microbiology* **6**, 2539-2548 (1992).

9 Liu, X. *et al.* Identification of Chlamydia trachomatis outer membrane complex proteins by differential proteomics. *Journal of bacteriology* **192**, 2852-2860 (2010).

10 Pais, S. V., Milho, C., Almeida, F. & Mota, L. J. Identification of novel type III secretion chaperone-substrate complexes of *Chlamydia trachomatis*. *PloS one* **8**, e56292 (2013).

11 Sisko, J. L., Spaeth, K., Kumar, Y. & Valdivia, R. H. Multifunctional analysis of *Chlamydia*-specific genes in a yeast expression system. *Molecular microbiology* **60**, 51-66 (2006).

12 Hower, S., Wolf, K. & Fields, K. A. Evidence that CT694 is a novel Chlamydia trachomatis T3S substrate capable of functioning during invasion or early cycle development. *Molecular microbiology* **72**, 1423-1437 (2009).

13 Weber, M. M., Bauler, L. D., Lam, J. & Hackstadt, T. Expression and localization of predicted inclusion membrane proteins in *Chlamydia trachomatis*. *Infection and immunity* (2015).

14 Qi, M. *et al. Chlamydia trachomatis* secretion of an immunodominant hypothetical protein (CT795) into host cell cytoplasm. *Journal of bacteriology* **193**, 2498-2509 (2011).

15 Chen, Y. S. *et al.* The *Chlamydia trachomatis* Type III Secretion Chaperone Slc1 Engages Multiple Early Effectors, Including TepP, a Tyrosine-phosphorylated Protein Required for the Recruitment of CrkI-II to Nascent Inclusions and Innate Immune Signaling. *PLoS pathogens* **10**, e1003954 (2014).

16 Belland, R. J. *et al.* Genomic transcriptional profiling of the developmental cycle of Chlamydia trachomatis. *Proceedings of the National Academy of Science of the United States of America* **100**, 8478-8483 (2003).

17 da Cunha, M. *et al.* Identification of type III secretion substrates of *Chlamydia trachomatis* using *Yersinia enterocolitica* as a heterologous system. *BMC Microbiology* **14**, 1-14 (2014).

18 Mirrashidi, K. M. *et al.* Global Mapping of the Inc-Human Interactome Reveals that Retromer Restricts Chlamydia Infection. *Cell host & microbe* **18**, 109-121 (2015).

19 Rzomp, K. A., Moorhead, A. R. & Scidmore, M. A. The GTPase Rab4 interacts with *Chlamydia trachomatis* inclusion membrane protein CT229. *Infection and immunity* **74**, 5362-5373 (2006).

20 Nunes, A. *et al.* Comparative expression profiling of the Chlamydia trachomatis pmp gene family for clinical and reference strains. *PloS one* **2**, e878 (2007).

21 Clifton, D. R. *et al.* A chlamydial type III translocated protein is tyrosine-phosphorylated at the site of entry and associated with recruitment of actin. *Proceedings of the National Academy of Sciences of the United States of America* **101**, 10166-10171 (2004).

22 Wu, X. *et al.* The chlamydial periplasmic stress response serine protease cHtrA is secreted into host cell cytosol. *BMC Microbiology* **11**, 87 (2011).

23 Hobolt-Pedersen, A. S. *et al.* Identification of Chlamydia trachomatis CT621, a protein delivered through the type III secretion system to the host cell cytoplasm and nucleus. *FEMS immunology and medical microbiology* **57**, 46-58 (2009).

24 Gong, S. *et al.* Chlamydia trachomatis secretion of hypothetical protein CT622 into host cell cytoplasm via a secretion pathway that can be inhibited by the type III secretion system inhibitor compound 1. *Microbiology* **157**, 1134-1144 (2011).

25 Caldwell, H. D. & Judd, R. C. Structural Analysis of Chlamydial Major Outer Membrane Proteins. *Infection and immunity* **38**, 960-968 (1982).

26 Le Negrate, G. *et al.* ChlaDub1 of Chlamydia trachomatis suppresses NF-kappaB activation and inhibits IkappaBalpha ubiquitination and degradation. *Cellular microbiology* **10**, 1879-1892 (2008).

27 Faal, N. *et al.* Temporal cytokine gene expression patterns in subjects with trachoma identify distinct conjunctival responses associated with infection. *Clinical and Experimental Immunology* **142**, 347-353 (2005).

28 Faal, N. *et al.* Conjunctival FOXP3 expression in trachoma: do regulatory T cells have a role in human ocular *Chlamydia trachomatis* infection? *PLoS medicine* **3**, e266 (2006).

29 Faal, N. *Conjunctival Immune Responses In Human Ocular Chlamydial Infections*, Open University, UK, (2011).

30 Burton, M. J. *et al.* Cytokine and fibrogenic gene expression in the conjunctivas of subjects from a Gambian community where trachoma is endemic. *Infection and immunity* **72**, 7352-7356 (2004).

31 WHO simplified trachoma grading system. *Community eye health / International Centre for Eye Health* **17**, 68-68 (2004).

32 Patton, D. L. *et al.* Whole genome identification of *C. trachomatis* immunodominant antigens after genital tract infections and effect of antibiotic treatment of pigtailed macaques. *Journal of proteomics* **108**, 99-109 (2014).

33 Kroll, T. C. & Wolfl, S. Ranking: a closer look on globalisation methods for normalisation of gene expression arrays. *Nucleic acids research* **30**, e50 (2002).

34 Magurran, A. E. in *Measuring Biological Diversity* 256 (Blackwell Science, 2004).

35 Gotelli, N. J. & Chao, A. in *Encyclopedia of Biodiversity* Vol. 5 (ed S. Levin) 195-211 (Academic Press, 2013).

36 Morris, E. K. *et al.* Choosing and using diversity indices: insights for ecological applications from the German Biodiversity Exploratories. *Ecology and Evolution* **4**, 3514-3524 (2014).

37 Last, A. R. *et al.* Plasmid Copy Number and Disease Severity in Naturally Occurring Ocular *Chlamydia trachomatis* Infection. *Journal of clinical microbiology* **52**, 324-327 (2014).

38 Roberts, C. H. *et al.* Development and Evaluation of a Next-Generation Digital PCR Diagnostic Assay for Ocular *Chlamydia trachomatis* Infections. *Journal of clinical microbiology* **51**, 2195-2203 (2013).

39 Christiansen, M. T. *et al.* Whole-genome enrichment and sequencing of *Chlamydia trachomatis* directly from clinical samples. *BMC infectious diseases* **14** (2014).

40 Andrews, S. FastQC: a quality control tool for high throughput sequence data. (2010).

41 Li, H. & Durbin, R. Fast and accurate short read alignment with Burrows-Wheeler transform. *Bioinformatics* **25**, 1754-1760 (2009).

42 Li, H. *et al.* The Sequence Alignment/Map format and SAMtools. *Bioinformatics* **25**, 2078-2079 (2009).

43 Danecek, P. *et al.* The variant call format and VCFtools. *Bioinformatics* **27**, 2156-2158 (2011).

44 Camacho, C. *et al.* BLAST+: architecture and applications. *BMC bioinformatics* **10**, 421 (2009).

45 Edgar, R. C. MUSCLE: a multiple sequence alignment method with reduced time and space complexity. *BMC bioinformatics* **5**, 113 (2004).

46 Gouy, M., Guindon, S. & Gascuel, O. SeaView version 4: A multiplatform graphical user interface for sequence alignment and phylogenetic tree building. *Molecular Biology & Evolution* **27**, 221-224 (2010).

47 Kearse, M. *et al.* Geneious Basic: an integrated and extendable desktop software platform for the organization and analysis of sequence data. *Bioinformatics* **28**, 1647-1649 (2012).

48 Vilella, A. J., Blanco-Garcia, A., Hutter, S. & Rozas, J. VariScan: Analysis of evolutionary patterns from large-scale DNA sequence polymorphism data. *Bioinformatics* **21**, 2791-2793 (2005).

49 Holsinger, K. E. *Tajima's D, Fu's F_S, Fay and Wu's H, and Zeng et al.'s E* (Stanford, California, USA, 2012).

50 Tajima, F. Statistical Method for Testing the Neutral Mutation Hypothesis by DNA Polymorphism. *Genetics* **123**, 585-595 (1989).

51 Fay, J. C. & Wu, C.-I. Hitchhiking Under Positive Darwinian Selection. *Genetics* **155**, 1405-1413 (2000).

52 Voight, B. F., Kudaravalli, S., Wen, X. & Pritchard, J. K. A map of recent positive selection in the human genome. *PLoS Biology* **4**, e72 (2006).

53 Nicholson, T. L. *et al.* Global Stage-Specific Gene Regulation during the Developmental Cycle of *Chlamydia trachomatis*. *Journal of bacteriology* **185**, 3179-3189 (2003).

54 Yu, C. S., Lin, C. J. & Hwang, J. K. Predicting subcellular localization of proteins for Gram-negative bacteria by support vector machines based on n-peptide compositions. *Protein Science* **13**, 1402-1406 (2004).

55 Yu, N. Y. *et al.* PSORTb 3.0: improved protein subcellular localization prediction with refined localization subcategories and predictive capabilities for all prokaryotes. *Bioinformatics* **26**, 1608-1615 (2010).

56 Goldberg, T. *et al.* LocTree3 prediction of localization. *Nucleic acids research* **42**, W350-355 (2014).
